# Antimicrobial Treatment Challenges in the Management of Infective Spondylodiscitis Associated with Hemodialysis: A Comprehensive Review of Literature and Case Series Analysis

**DOI:** 10.3390/antibiotics13030284

**Published:** 2024-03-20

**Authors:** Ioana A. Ratiu, Corina F. Moisa, Laura Țiburcă, Edy Hagi-Islai, Anamaria Ratiu, Gabriel Cristian Bako, Cristian Adrian Ratiu, Liana Stefan

**Affiliations:** 1 Faculty of Medicine and Pharmacy, University of Oradea, 1st December Square 10, 410073 Oradea, Romania; ioana.ratiu@didactic.uoradea.ro (I.A.R.); tiburca.lauraelena@student.uoradea.ro (L.Ț.); gabriel.bako@didactic.uoradea.ro (G.C.B.); lantal@uoradea.ro (L.S.); 2 Nephrology Department, Emergency Clinical Hospital Bihor County, 12 Corneliu Coposu Street, 410469 Oradea, Romania; 3 Rheumatology Department, Emergency Clinical Hospital Bihor County, 12 Corneliu Coposu Street, 410469 Oradea, Romania; 4 Faculty of Dentistry, University of Medicine and Pharmacy “Iuliu Hatieganu” Cluj-Napoca, Victor Babeș Street 8, 400347 Cluj-Napoca, Romania; hagi.islai.edy@elearn.umfcluj.ro (E.H.-I.); ratiu.anamaria@elearn.umfcluj.ro (A.R.); 5 Faculty of Medicine and Pharmacy, Dentistry Department, University of Oradea, 1st December Square 10, 410073 Oradea, Romania; ratiu_cristian@yahoo.com

**Keywords:** antimicrobial treatment, infective spondylodiscitis, epidural abscess, hemodialysis, end-stage renal disease, empiric antibiotic treatment

## Abstract

Infective spondylodiscitis (ISD), the infection of vertebral bodies and surrounding tissues, is a rare complication with major impact on the long-term survival of hemodialysis (HD) patients. Although the most frequent etiology is *staphylococcal*, identifying these pathogens in blood cultures and biopsy cultures is often difficult. This paper aims to present suitable antibiotic combinations for the treatment of these patients, which is usually challenging in the case of an unidentified pathogen. We presented the therapies applied for 13 HD patients and 19 patients without chronic kidney disease (CKD), diagnosed with ISD between 2013 and 2023 in Bihor County. The percentage of positive blood cultures was low in both groups (30.78% HD vs. 15.78% non-HD). The average length of antibiotic therapy was 5.15 weeks in HD patients and 6.29 weeks in non-HD patients. The use of Carbapenem alone (e.g., Meropenem) for an average of 19.6 days for patients in HD when the pathogen was not identified has proven to be efficient in most cases, similarly to using Vancomycin and Fluoroquinolone/Cephalosporines in combination. Regarding the non-CKD patients, the use of Clindamycin in various combinations for an average of 30.3 days has proven to be efficient in more than 90% of cases of ISD with a nonidentified pathogen. Within 2 years after ISD was diagnosed, 12 of the 13 HD patients passed away, mainly due to cardiovascular causes. Unfortunately, there are no guidelines in the literature concerning the empiric treatment of ISD in the particular case of HD patients. Upon checking the literature on PubMed and Google Scholar, only 10 studies provided relevant data regarding ISD treatment for HD patients. More data about the treatment and evolution of these patients is needed in order to elaborate a truly relevant metanalysis.

## 1. Introduction

Infective spondylodiscitis (ISD) is the infection of the vertebral bodies and the surrounding tissues including the intervertebral disc, with possible secondary development of osteomyelitis, vertebral body destruction and paravertebral abscess formation. The most frequent etiology is bacterial. Bacterial infection spreads hematogenously, usually adhering to a pre-existing vertebral lesion. The ISD incidence in HD patients reported in recent years has been continuously increasing, and it was cited to affect 4–28 patients/1 million people/year [1].

Compared to the general population, patients in hemodialysis (HD) have a series of predisposing factors for developing ISD, such as an immunosuppressed status, pre-existing bone/vertebral modifications due to mineral bone disease and age, high risk of bacteriemia secondary to manipulation of vascular access or associated with blood exposure to the extracorporeal circuit, and comorbidities.

End-stage renal disease (ESRD) affects both innate and adaptive immunity. All elements of innate immunity are affected, from the cellular receptors’ response to different stimuli to cellular dysfunction and cytokine explosion. The alteration of adaptive immunity in ESRD is caused by impaired activation of T lymphocytes, reduced numbers of B lymphocytes and antigen-presenting cell dysfunction [2,3,4]. The pro-inflammatory status in HD can be related to many conditions, including catheter or fistula infections, bioincompatible dialysis membranes, dialysate, endotoxin exposure, back filtration, chronic infections, and superimposed malnutrition [5]. Anemia due to erythropoietin deficit and blood loss during hemodialysis is also frequent. Therefore, the common inflammation biomarkers like nonspecific erythrocyte sedimentation rate (ESR) and C reactive protein (CRP) are often increased in HD without an obvious explanation [6], predicting all-cause and cardiovascular mortality [7,8].

Mineral bone disease encompasses two entities: fibrous osteitis (which can evolve with increased bone turnover) and adynamic bone disease (which consists of parathyroid hormone (PTH) over-suppression with low bone turnover). There are several chronic kidney disease—mineral bone disorder (CKD–MBD) biomarkers modified in ESRD [9]. Bone fragility associated with these conditions promotes the development of vertebral spine lesions under circumstances of minor trauma. These conditions are frequently associated with spondylodiscitis. A background of osteoporosis/osteopenia in chronic kidney disease (CKD) often leads to the association of commonly reversible destruction of the vertebra involved in the infectious-inflammatory process. High-flux hemodialysis has a decreased efficiency in the clearance of ß2-amyloid, which can deposit in all articular structures, including disks and intervertebral ligaments, making them more fragile [10,11]. Therefore, at least theoretically, hemodiafiltration (HDF) would be an effective method of reducing the incidence of ISD due to the higher clearance of ß2 amyloid [12,13,14,15]. However, we did not find relevant studies demonstrating the superiority of HDF in reducing the incidence of ISD in HD patients [16,17]. On the other hand, HDF increases the risk of infection, as additional filters and tubing are used for substitutive fluid.

**Figure 1:**
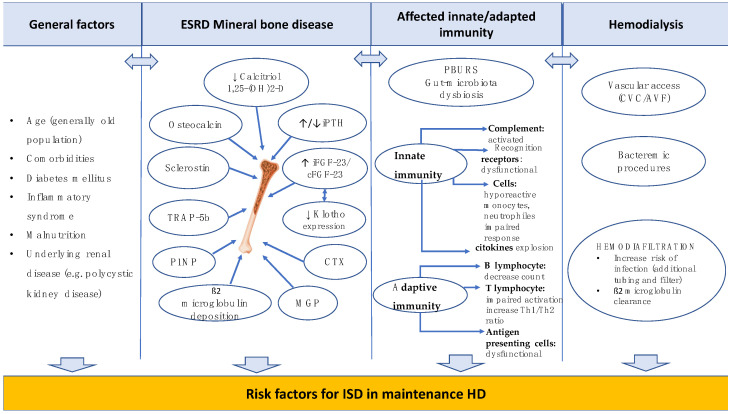
**The main risk factors associated with the development of ISD in hemodialysis patients.** Abbreviations: ESRD—end-stage renal disease; iPTH—intact parathormone; iFGF-23—fibroblast growth factor-23 intact; cFGF—c-terminal fragment fibroblast growth factor-23; CTX—Carboxy-terminal cross-linking telopeptide of type 1 collagen; TRAP-5B—Tartrate-resistant acid phosphatase isoform 5b; P1NP— intact-Procollagen type 1 N-terminal propeptide; MGP—matrix Gla protein; PBURS—protein-bound uremic retention solutes; CVC—central venous catheter; AVF—arteriovenous fistula, disease; ISD—Infective spondylodiscitis; HD—hemodialysis [3,4,5,9,12].

Bacterial inoculation of paravertebral disks occurs during bacteriemia or in cases of proximal infections (frequently urinary tract infections) [18]. The risk of bacterial hematogenous dissemination is increased in HD patients. Regardless of the venous access, punction of the AVF or the manipulation of CVC, the risk of bacteriemia is increased. However, most studies agree that manipulating CVC correlates with a higher incidence of bacteriemia compared to the use of AVF [19] (Figure 1).

Lacking associated specific symptoms, spondylodiscitis is a chameleon among the infectious diseases. The clinical picture of ISD includes dorsal/lumbar pain associated or not with fever and possible motricity modifications in the territory innervated by the nervous roots adjacent to the inflammation site [20]. Therefore, ISD might be diagnosed in the late stages [18].

According to Infectious Diseases Society of America (IDSA), the strongest criteria for ISD diagnosis encompass a new worsening back or neck pain with fever, increase in ESR or CRP, and presence of bloodstream infection. Thereupon, the appropriate diagnostic evaluation requires neurologic examination, blood cultures (two sets) and CRP, MRI or a combination with spine gadolinium Tc-99 bone scan, CT scan or positron emission tomography. The image-guided aspiration biopsy is recommended if the blood cultures are negative. IDSA advocates for surgical intervention in cases of progressive neurologic deficits and pain persistence despite appropriate AB therapy [21].

In clinical practice, in the absence of “red flags” symptoms, the diagnosis is established using laboratory and imaging methods. The gold standard is the magnetic resonance imaging (MRI) scan. In ISD, the inflammatory syndrome is constantly present. However, in HD patients, inflammation is frequent and, therefore, non-specific, correlated with multiple conditions mentioned above. These factors activate the innate immune system: increased CRP, leptins, C Cystatin, amyloid P, adiponectin, etc. [22,23]. The development of spondylodiscitis is a long-term and low-amplitude process that contributes to the subliminal, silent but permanent stimulation of the inflammatory response.

The most used laboratory tools for ISD diagnosis are the CRP level, increased in over 90% of the cases [24,25], blood cultures and cultures from the abscess biopsy. ESR (erythrocyte sedimentation rate) is non-specific [26,27], and pro-calcitonin has a minor role as a parameter of sepsis in the primary ISD [28].

Regarding the bacteriologic diagnosis, usually two blood culture sets (both containing one aerobic and one anaerobic bottle) are needed. The rate of positive results ranges between 25–59% but can rise up to 70% when the patient has not been previously treated with antibiotics. Moreover, the source of infection can be found in less than 50% of the cases [24].

The biopsy obtained from the abscess is the most reliable method for the pathogen identification, with a detection rate of up to 90% [29]. PCR and species-specific PCR can be used as well, to increase the detection rate. However, in this case, the sensitivity to antibiotics cannot be determined [30].

The MRI reveals discal lesions that may be associated with abscesses and destruction of adjacent bone tissue and gives information about the site and extension of the lesions [31,32].

Using these laboratory and imaging parameters, scales for diagnosis and treatment of ISD were elaborated (e.g., SponDT with IIA evidence level), but the extent of their application in real-life clinical practice is reduced [33].

The treatment of spondylodiscitis is a real challenge for a clinician. Since a microbial agent causes the inflammatory process, the therapy is mainly antibiotic, followed, in specific conditions, by a surgical drainage of the vertebral abscesses or reconstruction of the vertebral spine [34].

The antibiotic therapy should be directed at the etiologic pathogen; however, in most cases, the etiologic pathogen is not identified. Therefore, empiric schemes in terms of length and antibiotic choice need to be used [35].

The main consequence of an inappropriate antimicrobial selection would be the development of antibiotic resistance, one of the main problems that the medical field is dealing with at the moment [36].

The indications for surgical intervention are compression of neural elements, presence of epidural abscess, significant bone destruction with spinal instability, severe kyphosis, and failure of conservative management. Also, radiologically guided percutaneous drainage offers an effective alternative to surgery in the management of paravertebral and intradiscal abscesses. The spinal cord decompression is a surgical emergency, advisable in the first 24–36 h. However, neurological improvement after spinal cord decompression was registered even in patients with prolonged paralysis [37].

Therefore, given the experience we gained, we aimed to point out the antibiotic combinations we used for unidentified pathogens, the duration of the treatment and the therapeutic efficiency. We consider these data to be extremely useful in guiding practitioners to establish an adequate therapeutic strategy for patients in HD. These data can serve as a prerequisite for further studies. Moreover, in corroboration with the results of similar studies, the data in our study can be used in a meta-analysis, with actual statistical power regarding the optimal antibiotic combination and therapy length for these patients.

We divided our work into two parts: first, we presented our personal contribution regarding the antibiotics used in ISD in HD patients, and then we reviewed the literature.

## 2. Part I—Clinical Research

### 2.1. Materials and Method

The aim of this article is to improve the existing statistics regarding the patients in HD with ISD. We assessed the evolutive characteristics of these patients and the efficiency of different antibiotic combinations used, mainly empirical, mentioning the dynamics of the main inflammatory markers compared to the population with ISD without renal failure.

This observational, retrospective, noninterventional study included all patients who were admitted for ISD between 2013 and 2023 in the Emergency Clinical Hospital Bihor County, Nephrology and Infectious Diseases Departments. The study was conducted according to the guidelines of the Declaration of Helsinki and approved by the Ethics Committee of Emergency Clinical Hospital Bihor County (protocol no.1848/19.01.2024).

### 2.2. Diagnosis Criteria

The diagnosis criteria included (1) back pain, (2) increased inflammatory biomarkers, especially CRP, and (3) suggestive magnetic resonance imaging (MRI). This is in accordance with IDSA recommendations for the diagnosis and treatment of ISD. Out of 2157 patients undergoing hemodialysis in three different ambulatory centers, using the documents recorded during hospitalization, we identified 13 cases of ISD. Another 19 patients diagnosed with ISD had no renal insufficiency and were selected to form the control group. Most patients were included before the COVID-19 pandemic.

### 2.3. Hemodialysis Treatment Characteristics

The HD treatment was carried out in three maintenance HD centers even in the case of the patients with ISD, but hemodynamically unstable patients or those in need of spine immobilization received dialysis in the hospital’s hemodialysis department. The water purifying stations supplied pure water with under 0.1 UFC/mL. The dialyzers were not reused in any of the cases. Polysulfone membrane dialyzers were used, 1.9–2.1 m^2^, (ultrafiltration coefficient) Kuf 75–82 mL/h/mmHg, gamma-ray sterilization and helix one, 1.8–2.2 m^2^, UF coefficient 53–68 mL/h/mmHg, inline steam sterilization, at 121 degrees Celsius, 15 min. Hemodialysis prescriptions aim to achieve a kT/V value between 1.2–1.4 with three HD sessions per week. All of the patients in the HD program were evaluated according to the nationally approved protocols, monthly regarding complete blood count, urea, creatinine, electrolytes test, calcium, phosphorus, transaminases post-dialysis blood urea, and semestrially regarding iPTH, viral hepatitis markers, HIV, lipids, carbohydrate and protein metabolism, inflammation markers (CRP), and staphylococcal carriage.

### 2.4. Vascular Access Management in Hemodialysis Patients

For all HD patients, vascular access management was carried out according to the specific HD center protocols. In the presence of CVC, betadine or mupirocin ointments were applied on the exit site. When bacteremia due to CVC manipulation was suspected, two blood culture sets were obtained, and the empiric antibiotic therapy with vancomycin, 30 mg/kg loading dose associated with ceftazidime or ceftriaxone 2 g after HD was administered. In addition, HDF was suspended in these circumstances, and high-flux hemodialysis was started. Afterwards, the antibiotic therapy was adapted according to the antibiogram (if any). For hemodynamically unstable patients (with tunellitis and persistent fever after 48–72 h despite antibiotic therapy), the CVC was removed. In such situations, antibiotic therapy was maintained for 6–8 weeks, and a new catheter was inserted, if possible, in a different site, after 48–72 h of treatment. In the case of uncomplicated CVC infections (hemodynamically stable patient, with no signs of infectious systemic dissemination and with rapid improvement after initiating the antibiotic therapy), the length of the treatment was 2 weeks, maintaining the same CVC.

Antimicrobial catheter hub devices (TEGO, Clearguard) were not used for the patients in the study.

### 2.5. Collected Data for the Two Groups of Patients

The demographic characteristics such as age and gender, along with the clinical history of diabetes mellitus, recurrent infections, chronic viral infections or previously documented discopathies were compared between the patients in maintenance HD and non-HD. The percentages of positive blood cultures and biopsy cultures were evaluated, mentioning the identified pathogen, the existence of recent bacteremic procedures, the site of ISD and the necessity of neurosurgical treatment, as recorded in the hospitalization files. For HD patients, we registered the type of renal disease, HD and its length, the vascular access, as well as specific parameters such as chronic or transient nasal carriage of *Staphylococcus aureus*, HD efficiency and iPTH level, data provided by the patients’ ambulatory hemodialysis center.

The inflammation biomarkers WBC, neutrophils, CRP, ferritin, red cell distribution width (RDW) and hemoglobin level were registered at diagnosis and 3 months later.

The antibiotic regimen used and its length were recorded for all of the patients, and the clinical evolution of each case was mentioned. Patients without etiological diagnosis even after surgical biopsy received empiric broad-spectrum antibiotic therapy. The intravenous therapy was administered during hospitalization in all cases. The conservative orthopedic treatment consisted of 24/7 immobilization with a rigid orthosis until complete infection healing. The surgical treatment included debridement of necrotic tissues, decompression of neurological structures and spine instrumentation and abscess drainage followed by biopsy culture sampling.

### 2.6. Statistical Analysis

For statistical analysis, SAS version 9.1 was used, including student *t* test for continuous variables as well as chi-test (χ^2^) and Fisher Exact Test for categorical variables. The continuous variables were mean or median and standard deviation (SD) or interquartile range (IQR), respectively. Frequency data are presented as counts and percentages. Finally, the therapeutic efficiency and the evolution of the patients were mentioned.

## 3. Results

The demographic characteristics (Table 1) did not point out statistically significant differences regarding age (7th decade, with an average of 61.9 years in patients with HD and 64.6 years in patients with no HD (*p* = 0.25)) or gender (*p* = 0.35), with the mention that spondylodiscitis was more frequent in male patients. Consequently, the groups were comparable from this point of view. The history of recurrent infections did not differ statistically in the two groups (*p*-value = 0.83). Diabetes mellitus was significantly more frequent in non-HD patients (*p* = 0.077). Chronic hepatitis B or C were significantly more frequent in HD patients. The percentage of positive blood cultures was reduced in both groups (30.78% HD vs. 15.78% non-HD). However, procedures with a high risk of inducing bacteremia were present in similar percentages in the two groups, such as dental procedures and CVC insertion related in HD patients vs. drainage of abscesses in the second group. There were no significant differences regarding the history of discitis before the diagnosis of ISD in the two groups (*p* = 0.35). We found significant differences regarding the site of the inflammatory process: lumbar (69.23%) in HD patients and thoracic (73.68%) (*p* = 0.016), with three cases of paraparesis in the lastly mentioned group. The neurosurgical procedures were twice more frequent in non-HD patients (Table 1.)

While analyzing the group of patients in the HD program, we noticed that the etiological conditions of renal insufficiency were especially chronic glomerulonephritis, followed by vascular nephropathy and tubulointerstitial nephropathies. Therefore, the primary vascular injury appeared in 53.7% of cases. In the non-HD group, cardiovascular disease was diagnosed in 47.36% of patients (stroke + ischemic cardiopathy), suggesting the implication of the poor vascular bed in the progression of these lesions. Table 2 presents the characteristics of the HD patients (Table 2).

High-flux HD was carried out in all patients, and no patient had HDF. The vascular access was primarily an arteriovenous fistula (AVF) (69.23%, *p*-value was 0.04986, significant at *p* < 0.05). The average length of HD was 6 years, with differences regarding vascular access; 7.23 years in patients with AVF; and 2.3 years in patients with CVC. The average kTV was 1.38, the average PTH value was 332 pg/mL, and nasal staphylococcal carriage was documented in one patient (7.69%).

The clinical picture along with the paraclinical investigations at the onset and 3 months later in patients with or without CKD are presented in the tables below (Table 3 and Table 4).

Comparatively, the two groups had a significantly different behavior regarding the dynamics of the inflammatory parameters and anemia, correlated with the status of the ESRD patient (anemia, immunodepression, chronic inflammatory syndrome) (Table 5).

The antibiotic therapy was applied according to the identified bacterial pathogen and antibiogram, in collaboration with the infectious diseases specialists.

The HD patients received the following antibiotic treatment (Table 6).

The antibiotic treatments for the non-HD patients are detailed in Table 7.

The most efficient antibiotics for empiric treatment in HD patients are presented in the table below (Table 8).

## 4. Part II—Review of the Literature

This search aimed to present the therapeutic strategies used for the cases of ISD developed for patients in maintenance hemodialysis as reported in the literature, especially concerning the frequent situations of a nonidentified etiologic pathogen.

### 4.1. Materials and Methods

(*a*) *Data Sources*

Studies were identified by searching in two databases, Medline and Google Scholar. This search was made using two combined keywords. The first search was (spondylodiscitis AND hemodialysis) and the second one was (epidural abscess AND hemodialysis). Two different investigators collected the data independently, and finally, the results were compared.

The PRISMA statement was consulted throughout this review.

(*b*) *Inclusion Criteria*

The reports were included if they met the following criteria: (1) They refer to an ISD condition that occurred only in hemodialysis patients; (2) they include enough patients (at least four cases); (3) the studies describe the antibiotic treatment, preferably with details related to the type and duration of drug(s) administration; (4) the studies were published in English. We aimed to select case series, cross-sectional, prospective, retrospective, case-control or cohort studies.

### 4.2. Research Results

**Figure 2:**
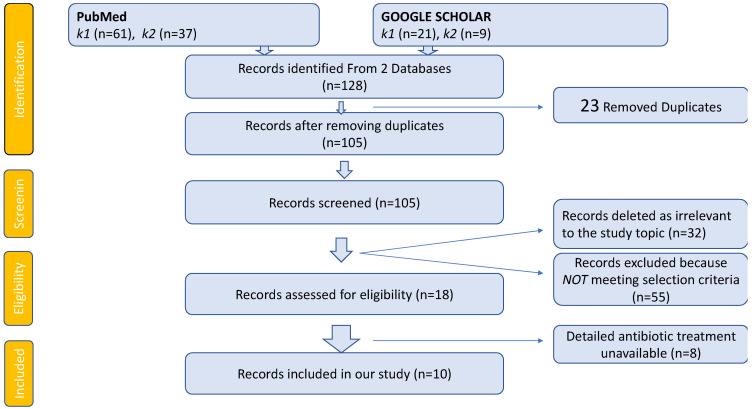
**PRISMA flowchart for our systematic review. Legend:** k1 (1^ST^ SEARCH) = keywords: spondylodiscitis AND hemodialysis; k2 (2^ND^ SEARCH) = keywords: epidural abscess AND hemodialysis.

Using PubMed and Google Scholar, we identified 128 articles containing the above-mentioned combined keywords. For the search using Google Scholar, we filtered the results according to the presence of the keywords only in the articles’ title. Twenty-three were duplicates and were removed from our list. Then, we checked each remaining study and found 55 case reports that included less than four patients, therefore, being excluded. We examined the rest of the studies and excluded any when the data were irrelevant to our topic. Finally, ten reports fulfilled the criteria mentioned earlier (Figure 2).

The table below lists the relevant selected studies (Table 9).

There are few studies with a small number of patients in the literature regarding this issue. Even though we identified 18 studies, only 10 articles were able to provide extensive data for our research (Figure S1, Supplementary Data). We only found case series relevant to our subject of study.

There is a higher amount of data concerning ISD in the general population than in ESRD patients. Gentile (2019) published a meta-analysis of 25 different studies, including 1756 patients. Microbiology data were available for 1060 cases, with *Staphylococcus* spp. (40.3%) and *Mycobacterium Tuberculosis* (30.9%) being the main etiologies. Of these cases, 27.8% were associated with neurological compromise, in 30.4%, the patients developed an abscess, and in 54.7%, the patients underwent surgery However, the antibiotic treatment was not specified [54].

Kuo G (2018) conducted a case–control study over 13 years including 105 ESRD patients treated by HD and 197 patients without CKD. Hemodialysis patients seemed to have reduced occurrence of fever, longer hospitalization, increased mortality and unfavorable outcome after 1 year compared to the other group. They found a correlation of ISD with the infection of the vascular access involving *MRSA*. Therefore, they recommend empiric AB therapy in any suspicion in patients on HD [40].

Regarding the patients with ESRD, Cervan et al.’s study, carried out for 23 HD patients diagnosed with spondylodiscitis, does not state the therapeutical schemes used in cases of identified vs. nonidentified etiology. It only specifies the type of therapy (empiric or definitive) and its length [38]. Lu Ya (2017) mentions the identified germs and their therapy for all 18 patients diagnosed with ISD. In case the germ was not isolated, it mentions the empiric therapy, its length and results. In some cases, surgical treatment of the paravertebral lesions was carried out [39].

In 2019, Madhavan K. identified 219 patients diagnosed with acute spondylodiscitis in an HD program in a systematic review; four patients were added from his own follow-up. He demonstrates that *Staphylococcus aureus* is the causative agent but does not mention the therapeutical schemes used; however, he mentions that antibiotic treatment was solely used in 76.8% of patients, and a quarter of the total number of patients studied needed to undergo surgical treatment as well [19].

Traversi et al. (2020) published a study after registering nine patients in an HD program diagnosed with ISD for 14 years. They mention that fever occurred in a small percentage of patients, 30% of patients showed neurological signs, and 90% of patients experienced pain. The main etiologic pathogen was *Staphylococcus aureus*, although it has been identified in less than 50% of total cases. The etiologic pathogen was not identified in the remaining patients. The antibiotic therapy used teicoplanin plus ciprofloxacin, administered on average for 6 weeks. However, depending on the case, they recommend extending the therapy for 6–12 weeks. There were no deaths reported; however, a patient had a second episode of ISD 2 years later, which led to neurological sequalae. In 2022, a study published in Surgical Neurology International evaluates the evolution of 11 patients in an HD program who had been diagnosed with ISD. The antibiotic treatment or its duration is not mentioned, but the study refers primarily to the surgical component [41].

Marco A, Ramirez Huaranga (2013) carried out a retrospective study for 4 years, during which five patients were diagnosed with ISD. Antibiotic treatment was used in all cases, and surgical procedures were unnecessary. The recommended therapy was intravenous antibiotic treatment for 4–6 weeks, followed by oral antibiotic treatment for 12–24 more weeks. The initial empiric antibiotic therapy should cover *Gram-negative* bacteria and *MRSA*, consisting of vancomycin associated with fluoroquinolone and cephalosporin [42].

Aydin Unal (2017) published a study regarding metastatic infections in patients in HD programs. Nine out of the 19 patients were diagnosed with ISD. In four cases, the treatment was antibiotic and surgical as well. The etiologic pathogen was identified in five cases: *Staphylococcus epidermidis*. The antibiotic treatments were either empiric or adapted to the antibiogram. The length of the antibiotic therapy should have been mentioned [43].

Cassó-Troche LR (2022) identified 11 patients with ISD in the HD program. The treatment used combined antibiotic therapy with surgical procedures. Five cases had recurrent infections. Blood cultures were negative in five cases, and in four cases, *staphylococcus aureus* was identified. The therapeutic schemes used were not mentioned [18]. Abid S (2008) presented 13 ISD cases in HD program patients. Of the blood cultures, 77% were positive, predominantly for *Staphylococcus aureus*. Antibiotic therapy was used for 6–12 weeks or until death. Surgical procedures were carried out in two cases. The mortality rate was 46% [44].

Chen, LH (2010) referred to the surgical treatment of discitis for 16 patients in HD. The indications for the treatment were drainage of the abscess, an etiologic diagnosis, treatment of infections refractory to non-surgical therapies, decompression of neural elements in the presence of a neurological deficit, and correction of spinal deformity or instability. The bacterial pathogen was identified and represented by *Staphylococcus aureus* MSSA (methicillin-sensitive *Staphylococcus aureus*) in 11 patients and MRSA (methicillin-resistant *Staphylococcus aureus*) in four patients [45]

Wong presented six patients in HD out of nineteen patients diagnosed with ISD. The etiologic pathogen was mentioned: *MRSA* in two cases, *MSSA* in the other two, and in two cases, the pathogen was not identified. The duration of antibiotic therapy was 6.5 weeks, without mention of the type of medication used [46].

Kovalik (1996) identified in a retrospective study 10 HD patients diagnosed with ISD, eight of them with CVC and two with a synthetic graft. He mentioned the necessity of surgical abscess drainage in six patients, associated with the intravenous administration of 1–1.5 g Vancomycin to achieve the therapeutic drug level [47].

Vinay Jain (2020) published a study including 34 patients in an HD program with ISD. The etiologic agents were found in 28 cases from the biopsy culture: *MRSA* (38.2%), *MSSA* (11.7%), *Staphylococcus epidermidis* (5.8%), *Streptococcus* (11.7%), *Enterococcus* (8.8%), and *Mycobacterium tuberculosis* (5.8%). The antibiotic treatment and its length were not specified [48].

Mei-Wu (2011) compared the evolution of the patients with ISD with and without ESRD. The identified etiologic agents were: *Staphylococcus aureus* (41.7%), oxacillin-sensitive *S. aureus* (25%), oxacillin-resistant *S. aureus* (16.7%), and *Enterococcus* (16.7%). Of these patients, 41.47% exhibited no growth. The antibiotic treatment was not mentioned [49].

In 2018, Lu-Ya published a study regarding the epidemiology and the outcome of patients with ISD and HD. No data regarding the antibiotic treatment were recorded [50].

Yildirim S. (2022) refers to 15 patients with ISD secondary to CVC infection. He concluded that the only factor associated with resistance to medical treatment was the period between admission and diagnosis. There were no data regarding antibiotic treatment [51].

Tsuchiya (2001) identified nine patients with ISD and HD, but referred retrospectively to the onset of infection, characteristics of clinical symptoms and evolution, with no information about the treatment applied [52].

Faria (2011) presented the outcome of 11 HD patients with ISD. Blood cultures were positive in all patients, and *Staphylococcus aureus* was identified in eight cases. Ten patients had a CVC for hemodialysis access, and the number of vascular accesses in their medical histories was higher than in the rest of the HD population. Four patients (36%) died during follow-up. None of the patients who underwent vancomycin and gentamicin antibiotic therapy died. In conclusion, prolonged antibiotic therapy with initial broad-spectrum coverage seemed to be the best therapeutic approach [53].

Finally, from 18 studies identified, only ten articles were able to provide extensive data for our research (Figure S1, Supplementary Data).

In all previous studies, the antibiotic therapy was adapted to the identified bacteria (if any) (Table 10).

The great challenge was to treat an infection with an uncertain etiology. In these cases, combined antibiotics were used, with the results shown in Table 11.

In conclusion, in ESRD patients *MRSA* has been found as a major etiology in several studies performed [19,38,39,41,42,43,44,45,46,47,48,49,53]. Hemodialysis catheters and the period between diagnosis and admission were associated with resistance to medical treatment. Moreover, the number of vascular accesses seems to correlate with the risk of ISD.

Antibiotic treatment remains the most important therapy. It should be started whenever there is a suspicion of *MRSA* infection, initially based on empiric statistical data and consecutively based on laboratory results (blood cultures, antibiogram).

The duration of antibiotic administration varies between 6–12 weeks up to 24 weeks. Surgical treatment (drainage of abscesses) may be required in about a quarter of the patients (0–50%, depending of the study) [19,42,45,47].

## 5. Discussions

### 5.1. General/Demographic Considerations

Although ISD could be a possible cause of dorso-lumbar pain syndrome in HD patients, the lack of specificity of the clinical picture might lead to the underdiagnosis of this condition. The articles in the literature cite a series of ISD cases diagnosed in patients in HD programs, without registering enough patients to increase the statistical relevance of the carried-out studies [38,39,40,41,42,43,44,45,46,47,48,49,50,51,52,53,54,55,56]. In the present study, we identified 13 patients with ISD (about 0.6% out of the 2157 dialyzed over the last 10 years), less than the data in the literature [57].

Patients with ISD and ESRD are treated in chronic HD centers in our county. The centers currently have 100 HD and HDF devices, with approximately 500 patients included only in the ambulatory system. The vascular access is an AVF in 65% of cases, CVT in 30%, and temporary CVC in 5% in anticipation of the definitive access. We have no patients with AV grafts.

One of the predisposing factors for the occurrence of ISD is old age. The average age of the patients with ISD at the time of diagnosis was 61.92 years, with no statistical differences in our study from the non-HD patients (64.63 years, *p* = 0.25). The value is similar to that of other studies in the literature [58,59]. However, in some lower amplitude studies, the average age was higher [19]. In our research, the male gender appears to be a predisposing factor for ISD occurrence. Gender distribution regarding ISD is variable in the literature. Most studies reveal an increased incidence in male patients [60,61]. A possible explanation may be that the general risk factors for ISD, such as traumatic lesions of the spine, alcohol and drug abuse, and immunodeficiency are more frequent in male patients. On the other hand, the higher levels of estrogen in women seem to have an immune-modulating effect (e.g., expression of structures of the innate immunity), making female patients less prone to major infections and increasing their response to conservative or additional medical therapy in cases of systemic infection. However, features such as old age and menopause, which are also common for our patients, may lower the immunomodulatory effects of estrogens. Furthermore, a recent study evaluating ISD patients in HD demonstrated a higher incidence of ISD in female patients [62].

### 5.2. Associated Diseases

Although discordant with the literature [36] and unlike the population without CKD, the incidence of ISD in diabetic patients in the HD program was reduced. We did not evaluate the glycemic control of these patients. As our data were retrospectively collected, and as HbA1c is not a routine determination in HD centers, the value of this parameter was unknown for some diabetic patients. However, although HbA1c is considered the gold standard for glyco-metabolic monitoring, there are some setbacks when it comes to the patients in hemodialysis due to associated anemia, which can underestimate the HbA1c levels [63]. On the other hand, secondary to glucose removal during the dialysis session, our patients experience episodes of hypoglycemia more frequently during the intradialytic period. Such hypoglycemic episodes occur as well during the interdialytic period due to reduced renal clearance of insulin. Consequently, the glycemic monitoring of the HD patients through HbA1c or glycated albumin is unreliable because these parameters are significantly influenced by different variables that could both overestimate and underestimate these values [64]. Therefore, the daily capillary blood glucose levels are needed to guide possible adjustments of insulin dosing [65]. However, it is widely accepted that a value of HbA1c greater than 9% is correlated with an increased cardiovascular risk of patients in the HD program [66]. Regarding the cardiovascular risk, this parameter would have been useful for our patients, especially since all of the patients passed away due to cardiovascular causes within 2 years after being diagnosed with ISD.

The non-HD patients with ISD had an increased incidence of chronic viral hepatitis. It is a well-known fact that the chronic hepatitis viruses, especially B and C, induce complex alterations of innate and adaptive immunity. In chronic phases, the presence of hepatitis viruses causes changes in the cells involved in the immune response: dendritic cells (antigen-presenting cells), myeloid-derived suppressor cells (MDSCs), regulatory T cells, regulatory B cells, monocytes, macrophages, CD4, CD8, and natural killer cells, modifying the circulant cytokine profile [67].This immune imbalance can reduce the organism’s resistance to other microbial agents, promoting bacteriemia and inoculation of intervertebral disks on a defective background.

### 5.3. Microbiological Findings. Vascular Access

Intervertebral disk bacterial inoculation occurs most frequently through hematogenous dissemination from an infectious hotspot. The presence of a CVC is the leading cause cited in the literature regarding bacteremia in HD patients, septic metastases, as well as ISD [68,69,70].

The blood cultures were positive in a reduced number of cases at the time the ISD diagnosis was established; we found the presence *of Staphylococcus aureus* in two patients, *Staphylococcus epidermidis* in one patient (possible contamination) *and Providencia stuardii* in one patient. The negative results were due to the routine administration of antibiotic therapy when the clinical signs of bacteremia occurred (shivers, fever in HD with no other discernable cause, especially in patients with CVC), with no possibility of performing blood cultures. Furthermore, considering the ambulatory character of the HD service and the frail patients it addresses, prescribing antibiotic therapy was insufficiently justified in many cases. This can explain the existence of a subclinical bacteremia, with sufficient germs to inoculate the intervertebral disks.

In contrast with the data in the literature [53], in our study, ISD was diagnosed in a lower percentage of patients with CVC compared to those with AVF. The management of the AVF was carried out using the existing protocols in chronic HD centers. However, the care of the AVF at home, carried out by the patient, could not be documented. The CVC was inserted in two patients in the HD program, 3 months before the occurrence of ISD. The ISD occurrence in patients with AVF can be justified by their long history in the HD program (7.22 years as compared to 3.25 years in the case of CVC). Dental procedures were carried out in many patients, with no possibility of documenting the efficiency of antibiotic coverage in these situations. However, we identified a pulmonary and a urinary hotspot in patients with polycystic kidney disease. HDF is cited as a possible source of bacteremia due to the additional circuit of the substitution liquid, but this did not apply to our patients. The immune imbalances caused by ESDR and HD can also be involved in the occurrence of ISD in patients with AVF [2,3,71,72].

### 5.4. Clinical and Biological ISD Features

The infectious picture was subclinical and nonspecific: persistent and progressive paravertebral pain, invalidating, accompanied by functional impotence of the limbs and paralysis. Paraparesis was present in one case due to abscess dissemination in the spinal space. In this case, a surgical procedure was necessary for drainage and decompression. This was the only case requiring this procedure. In the literature, neurosurgical interventions are frequent [38,39]. Reconstructive strategies could be performed, and the use of PRGF in this context should be an option for HD patients [73]. The localization of the infectious process was predominantly lumbar in HD patients (61.63%); the data in the literature concerning this matter are divergent [17,39].

The diagnosis of ISD was tardy in all patients, after weeks of pain complaints treated symptomatically. The inflammatory markers were increased at the moment of diagnosis in both groups and significantly higher in HD patients (*p* = 0.0017 for CRP). Although we evaluated many inflammatory markers, CRP was the most accurate marker of the evolution. Unlike the general population, in HD patients, WBC are not always increased, while neutrophilia is still present. In HD patients with ISD, anemia generally worsens, due to the diminished response to erythropoietin administration, caused by inflammation. However, the parameter that is better correlated with ISD than plasma hemoglobin is the value of RDW: the higher the value of red cell distribution width (RDW), the greater the inflammation/infection of the disks. PTH, a marker of the osseous turnover, was increased in patients in HD, with no values out of control as we administered phosphate binders, vitamin D or selective vitamin D receptor activators. We used the MRI scan in all cases.

### 5.5. Therapeutic Strategies

The antibiotic regimen for the HD patients was based on Vancomycin administration (61.53% HD vs. 38.88% non-HD). The choice of Vancomycin in patients with negative cultures was based on the etiology most frequently reported in the literature [19,38,39,40,43]. Vancomycin was used for 2–4 weeks and was later replaced with other anti-*staphylococcal* agents (Clindamycin, Ciprofloxacin, Amikacin). Surprisingly, the use of Carbapenem alone (e.g., Meropenem) for an average of 19.6 days for patients in HD when the pathogen was not identified has proven to be efficient in most cases, similarly to using Vancomycin and Fluoroquinolone/Cephalosporines in combination, for an equal amount of time.

The carbapenems are potent, broad-spectrum antibiotics that have been shown to be safe and efficient in the treatment of serious infections. Their *Gram-negative* coverage, superior to that of other β-lactams, as well as their stability against extended-spectrum β-lactamases and AmpC β-lactamases makes them an effective weapon in the treatment of many multidrug resistant (MDR) bacteria [74,75]. Difficult-to-treat infections need to be cured with broad-spectrum antibiotics. Therefore, we are left with two options: either using a combination of drugs with the risk of adverse reactions or using one of the few possibilities of antibiotics in monotherapy, such as carbapenems. Several studies indicate that if carbapenems are used in proper conditions at early stages of infection and for a sufficient amount of time, the risk of developing carbapenem resistance is not justified [53]. So, the question remains: is ISD a difficult to treat infection? If ISD is present, with an unidentified pathogen and especially in the context of a hemodialysis patient, our response will definitely be positive.

Recently, advanced-generation cephalosporins, like ceftaroline-fosamil, have demonstrated efficacy in treating MRSA strains and ISD [76].

Patients without renal failure were treated with antibiotic regimens based on Clindamycin. The use of Clindamycin in various combinations for an average of 30.3 days has proven to be efficient in more than 90% of cases of ISD with a nonidentified pathogen. These data can serve as a prerequisite for further studies. Clindamycin is FDA-approved to treat septicemia, intra-abdominal infections, lower respiratory infections, gynecological infections and *Chlamydia trachomatis cervicitis*, recurrent *group A streptococcal* pharyngitis, bone, joint skin structure infections [77]. Clindamycin is used in soft tissue infections due to its efficacy against *MRSA*. Clindamycin is also a choice for outpatient treatment because of its cost, availability, and effectiveness against methicillin-resistant *Staphylococcus aureus* [78,79]. The combination with quinolones (e.g., ciprofloxacin) was frequently used. This combination increased the serum bactericidal action against *Gram-positive* strains (peak titers) of *Staphylococcus aureus* and *Streptococcus pneumoniae* [80]. Vancomycin remained a therapeutical option for this group of patients, even if the staphylococcal infection was not documented. The aminoglycosides, potent, broad-spectrum antibiotics, were added to clindamycin, meropenem or ciprofloxacin due to their excellent action against members of the *Enterobacteriaceae family, Staphylococcus* aureus, including methicillin-resistant and vancomycin-intermediate and -resistant isolates, *P. aeruginosa* and, to a lesser extent, *Acinetobacter baumannii.*

The average length of the antibiotic therapy was 5.15 weeks in HD patients and 6.29 weeks in non-HD patients, similar to the data in the literature [19], with favorable evolution of 61.53% cases (HD) and 84.21% (non-HD). The length of the antibiotic treatment was dictated by the dynamics of the inflammation and the clinical picture. It must be mentioned that the HD patients received other empirically administered antibiotics during the HD sessions before the antibiotic therapy for ISD treatment. This is a bias element in interpreting the data. Two years after ISD was diagnosed, 12 of the 13 patients in HD had passed away, mainly due to cardiovascular causes.

Antibiotic resistance is one of the paramount problems that the medical field is dealing with at the moment. Unfortunately, as we have previously mentioned, within 2 years following their ISD diagnosis, we lost most of the patients.

### 5.6. The Limitations of the Study

The limitations of this study are related to its retrospective, observational design, as well as the reduced number of included patients, strictly related to the decreased incidence of this disease in hemodialysis. Moreover, the poor long-term evolution of our patients hindered the evaluation of other parameters such as development of antibiotic resistance, which could be possible secondary to the increased length of antibiotic therapy for unidentified pathogens.

However, despite its limitations, our study aims to shed some light on this rare and challenging disease, ISD in hemodialysis, providing a possible direction for practitioners to follow in terms of diagnosis and treatment. Moreover, in corroboration with the results of similar studies, the data in our study can be used in a meta-analysis, with actual statistical power regarding the optimal antibiotic combination and length of therapy for these patients.

## 6. Conclusions

ISD is a rare infectious complication, but it has a significant impact on the long-term survival of HD patients. Although most studies indicate staphylococci as the major etiology, the actual identification of the pathogen in hemocultures and biopsy cultures is difficult for these patients. Due to the ISD risks associated with CVC, increasing the percentage of patients with AVF up to 80% in HD centers could reduce the incidence of ISD. If ISD is clinically suspected, HDF should be replaced with classic HD. The correct diagnosis is established by correlating the clinical picture with the values of inflammation markers and imaging-based findings. The broad-spectrum antibiotic therapy should mainly be based on anti-*staphylococcal* treatment with a combined antibiotic strategy in the absence of the etiologic factor. The duration of the therapy should be extended to approximately 4–6 weeks to ensure efficacy and to prevent the development of antimicrobial resistance. Finally, nephrologists should be aware of the higher risk of ISD in HD patients, making early diagnosis and treatment of these patients possible.

## Figures and Tables

**Table 1:** Patients’ main characteristics.

Patients’ Characteristics	Chronic HD (N = 13)	Non-HD Patients (N = 19)	*p*-Value, χ^2^
***Age* (*years*), *median* (*IQR*) **	63 (8)	66 (18)	0.252
***Male gender number* (*%*) **	10 (76.92)	16 (84.2)	0.355
***Recurrent infections* (*%*) **	8 (61.53)	11 (57.89)	0.836
***Diabetes mellitus* (*%*) **	1 (7.69)	7 (36.84)	0.077
***Chronic hepatitis C/B* (*%*) **	5 (38.46)	2 (15.38)	0.060 χ^2^ = 3.524.
***Positive blood culture* (*%*) **	4 (30.78)	3 (15.78)	0.149 χ^2^ = 2.076
***Previous documented*** ***discopathy* (*%*)**	4 (30.78)	7 (36.84)	0.355
***Recent bacteremic procedures* (*3 months before ISD*) ** - ***Vascular access insertion*** - ***Dental procedures*** - ***Abscess drainage***	2 7 -	- 9 3	
***Location ISD* (*MRI*) (*%*) ** - *thorax* - *lumbar*	4 (30.78) 9 (69.23)	14 (73.68) (3 paraparesis) 5 (26.31)	0.016 0.016
***Neurosurgical procedures* (*%*) **	1(7.69%)	4(21.05%)	0.306

IQR = interquartile range, χ^2^ = chi square test.

**Table 2:** Hemodialysis patient characteristics.

Hemodialysis Patient Characteristics		Number (%)	*p* Value
***1. Primary kidney disease***	Chronic glomerulonephritis Tubulointerstitial diseases Polycystic kidney disease Vascular nephropathy Diabetic nephropathy	4 (30.7) 3 (23) 2 (15.38) 3 (23) 1 (7.69)	
***2. Hemodialysis type:*** *High-flux hemodialysis*		13 (100)	
***3. Vascular access***	Long term catheter Arteriovenous fistula	4 (30.7) 9 (69.23)	0.049
***4. Duration of hemodialysis, mean* (*years*)** *Long-term catheter* *Arteriovenous fistula*		6 3.25 7.22	
***5. Hemodialysis efficiency* (*kT/V*) **		1.38	
***6. PTH*** (*pg/mL*)		332.3	
***7. Staphyloccocus nasal carriage***		1 (7.69)	

Abbreviations: iPTH = intact parathormone.

**Table 3:** Clinical-biological parameters—HD patients.

	Symptoms	Diagnostic Tools	CRP		WBC		Neutrophils		Hb		RWD	Ferritin	iPTH
			dgn	3M	dgn	3M	dgn	3M	dgn	3M	dgn	dgn	dgn
1	pain fever	**MRI T10-11**	148	42	14,000	7800	12,000	6000	10.5	9.3	16	440	200
2	pain, fever limb weakness	**MRI** **L2-L3**	150	39	9700	6200	7300	4900	9.5	9.4	17	720	320
3	pain	**MRI** **T12-L1**	35	12	5500	5100	3800	3200	12.2	12.4	18	410	120
4	fever limb weakness	**MRI** **L1-L3**	350	218	28,000	24,000	20,000	19,500	9.5	7.9	16.5	730	210
5	pain limb weakness	**MRI** **T10-T11**	81	228	8200	6600	5700	4500	10.2	11.3	16.9	530	419
6	pain, fever	**MRI** **L1-L2**	210	42	8500	8500	6200	6300	11.2	9	13.8	630	110
7	pain fever limb weakness	**MRICT** **L2-3**	227	65	18,500	8900	15,600	7000	6.6	9	20.4	1089	93
8	pain limb weakness	**MRI** **L4-l5**	290	11.5	12,000	6800	9900	4800	9.6	12.5	17.2	620	511
9	pain fever	**MRI** **L1-L2,** **L4-L5**	62	23	4100	4200	2600	2400	8	9.6	16.1	419	802
10	pain fever	**MRI** **D11-D12**	88	17	11,300	7300	7700	5200	10.5	11	15.5	520	340
11	pain fever	**MRI** **L2-L3**	161	11.6	19,300	12,100	15,700	8200	9,6	11.7	16.9	530	570
12	pain fever	**MRI** **T10-T11**	120	NP	12,000	NP	9800	NP	8.9	NP	15.3	470	265
13	pain fever	**MRI** **T7-T8**	150	25	18,000	6500	15,300	4200	10.2	11.5	14	520	360

Abbreviations: RDW—red cell distribution width, dgn—diagnosis, M—months, CRP—C reactive protein, WBC—white blood cells, Hb—hemoglobin, iPTH = intact parathormone.

**Table 4:** Clinical-biological parameters—non-HD patients.

	Symptoms	Diagnostic Tools	CRP		WBC		Neutrophils		Hb	
			dgn	3M	dgn	3M	dgn	3M	dgn	3M
1.	pain	**MRI**	56	4.5	9700	5200	7300	4200	13.6	14.5
2.	pain/limb weakness	**MRI**	130	NP	11,600	NP	10,000	NP	12.4	NP
3.	pain	**MRI**	48	3	7500	5300	6000	4900	14.5	14.7
4.	fever/limb weakness	**MRI**	56	17	7900	5300	6300	4200	13.6	14.2
5.	pain/limb weakness	**MRI**	120	37	9800	6500	N/A	N/A	12	11.9
6.	pain/hemiplegia	**MRI** **T11/12**	10.19	4.16	6100	7000	4000	5000	14.1	16
7.	pain/limb weakness	**MRI** **L2-l3**	161	6.4	16,000	9900	14,000	7100	12.3	12.9
8.	pain	**MRI** **L4-l5**	38	1.4	5700	N/A	2000	N/A	13.6	N/A
9.	pain	**MRI** **T10-11**	46	NP	7200	NP	4900	NP	13.3	NP
10.	pain	**MRI** **L5-S1**	1.7	12	10,200	8000	4600	N/A	14	15
11.	monoparesis	**MRI** **L2-S2 (abscess)**	22.7	3.5	8700	8300	4700	3400	12.2	12
12.	pain /paraparesis	**MRI** **T10-T11**	85	N/A	12,000	N/A	9800	N/A	12	13.5
13.	pain	**MRI** **T12**	95	N/A	18,000	N/A	16,000	N/A	12.3	N/A
14.	pain	**N/A**	113	61	7600	8400	4300	4900	10.2	10
15.	pain	**MRI** **L4-L5 (abscess)**	42	31	7500	10,200	5100	6300	11	12.7
16.	pain	**MRI** **L1-L2**	11	N/A	7100	N/A	4400	N/A	12.5	N/A
17.	pain	**N/A**	67	N/A	9700	N/A	6700	N/A	11	N/A
18.	pain	**N/A**	129	5.4	9200	N/A	6800	N/A	13	N/A
19.	paraplegia	**MRI** **T8-T9**	108	8	7400	4200	N/A	N/A	9.7	10.2

Abbreviations: RDW—red cell distribution width, dgn—diagnosis, M—months, CRP—C reactive protein, WBC—white blood cells, Hb—hemoglobin, iPTH = intact parathormone.

**Table 5:** Comparative analysis at the time of diagnosis and after 3 months.

Laboratory Biomarkers	CRP (Mean)		WBC		Neutrophils		Hb Level	
	dgn	3M	dgn	3M	dgn	3M	dgn	3M
**Chronic hemodialysis** **(mean)**	159.38	53.69	13,007	8578	10,123	6338	9.42	10.31
**Non-HD**	70.5	16.5	9415	7253	6876	5110	12.49	13.04
***p*-value** **ES (effect size)**	0.0005 0.001	0.03 0.01	0.02 0.0001	0.19 -	0.028 -	0.1 -	0.00001 0.09275	0.0001 0.08

Abbreviations: dgn—diagnosis, M—months, CRP—C reactive protein, WBC—white blood cells, Hb—hemoglobin.

**Table 6:** Antimicrobial therapy for patients in the hemodialysis program.

	Blood Culture	Culture from Biopsy	Other Cultures	Antimicrobial Therapy	AB Therapy Length	Outcome
1.	***Staphylococcus*** ***epidermidis***	(NP)	*E. coli* (uro culture)	Vancomycin + Ceftazidime, then Vancomycin + Meropenem then Ciprofloxacin + Clindamycin	2 w 4 w 6 w	recovered died 1 year later
2.	***Staphylococcus aureus***	(-)	NP	Vancomycin + Meropenem Clindamycin	3 w 2 w	died
3.	(-)	(NP)	NP	Meropenem	2 w	recovered
4.	(-)	(NP)	(-)	Meropenem	3 w	recovered
5.	(-)	(NP)	(-)	Vancomycin + Meropenem	3 w	died
6.	(-)	(NP)	NP	Meropenem	2 w	recovered
7.	(-)	(NP)	NP	Vancomycin + Cefoperazon	3 w	recovered
8.	***Staphylococcus aureus***	(NP)	NP	Cefrtiaxon + Amikacin Vancomycin	1 w 4 w	recovered
9.	(-)	(-)	NP	Vancomycin + Meropenem Clindamycin	4 w 4 w	recovered
10.	(-)	(NP)	NP	Clindamycin Cefixime	3 w 3 w	died
11.	***Providencia stuartii***	***Providencia stuartii***	NP	Ceftazidime/avibactam	8 w	died
12.	(-)	(NP)	NP	Cefoperazone/sulbactam Vancomycin + Ciprofloxacin Ciprofloxacin + Cefuroxime	2 w 2 w 2 w	died
13.	(-)	(NP)	NP	Vancomycin + Cefoperazone + Ciprofloxacin Clindamycin	2 w 2 w	recovered

Abbreviations: NP—not performed, (-)—negative, w—weeks. R—recovered, D—died.

**Table 7:** Antimicrobial therapy for non-HD patients.

Case No	Blood Culture	Other Cultures	Antimicrobial Therapy	AB Therapy Length	Outcome
1.	(-)	Uro Culture (-)	Clindamycin Ciprofloxacin	3 w 3 w	recovered
2.	***Staphylococcus*** ***aureus***	(NA)	Clindamycin + Amikacin	8 d	died
3.	(-)	(-)	Clindamycin + Ciprofloxacin	8 w	recovered
4.	(-)	(-)	Clindamycin + Ciprofloxacin		recovered
5.	(-)	*Staphylococcus* *Epidermidis*	Vancomycin + Amikacin Amoxicillin/clavulanate + Ciprofloxacin	2 w 3 w	recovered
6.	(-)	(-)	Clindamycin + Ciprofloxacin	6 w	recovered
7.	***E. coli***	*Coproculture* (*Candida* *Albicans*)	Clindamycin + Ciprofloxacin Vancomycin + Imipenem Cefoperazone/sulbactam	10 d 10 d 4 w	recovered
8.	***Staphylococcus*** ***epidermidis***	*Candida* *albicans*	Vancomycin + Ceftriaxone Ceftriaxone + Clindamycin	2 w 2 w	recovered
9.	(-)	(-)	Vancomycin + Ceftriaxone Ciprofloxacin + Clindamycin *then* Teicoplanin	10 d 10 d 2 w	died
10.	(-)	(-)	Vancomycin + Clindamycin Amoxicillin/clavulanate + Ciprofloxacin	6 w 2 w	recovered
11.	NP	*Candida* *albicans*	Ciprofloxacin + Amikacin Clindamycin + Ciprofloxacin	4 w 4 w	recovered
12.	NP	(-)	Clindamycin + Ciprofloxacin Amoxicillin/clavulanat Doxycycline	3 w 2 w 4 w	recovered
13.	(-)	(-)	Vancomycin + Ceftriaxone	2 d	died
14.	(-)	(-)	Clindamycin+Ciprofloxacin + Gentamicin Clindamycin	2 w 4 w	recovered
15.	(-)	(-)	Meropenem + Gentamicin Clindamycin	1 w 4 w	recovered
16.	(-)	(-)	Clindamycine + Gentamicin Clindamycin	3 w 3 w	recovered
17.	(-)	(-)	Amikacin + Clindamycin Clindamycin	2 w 4 w	recovered
18.	(-)	(-)	Clindamycin + Ciprofloxacin Clindamycin	3 w 3 w	recovered
19.	(-)	NP	Vancomycin	2 we	recovered

Abbreviations: NA—not available, (-)—negative, NP—not performed, d—days, w—weeks.

**Table 8:** The most efficient antibiotics for empiric treatment in HD.

HD Patients	Meropenem	Vancomycin	*p* Value
**Number of patients (%)**	5 (55.5%)	5 (55.5%)	-
**Length of treatment (days)**	19.6	19.6	-
**Recovered patients no (%)**	4 (80%)	3 (60%)	*0.0032*
**Monotherapy/** **Recovered patients**	3 (60%)/ 3 (100%)	-	*0.00001*
**Combined therapy/results**	2 (40%) Vancomycin + Meropenem, (died) Vancomycin + Meropenem, (recovered)	5 (100%) Vancomycin + Meropenem (died) Vancomycin + Cefoperazon (recovered) Vancomycin + Meropenem (recovered) Vancomycin + Ciprofloxacin (died) Vancomycin + Cefoperazone + Ciprofloxaci (recovered)	*0.00001*

**Table 9:** Relevant studies referring to spondylodiscitis in HD patients.

Nr.	Article	Number of Patients	Follow-Up Period
1	Cervan A.M., 2012 [38]	23	1996–2010 (14 years)
2	Lu Y.A., 2017 [39]	18	2005–2015 (10 years)
3	Kuo G., 2018 [40]	105	2002–2015 (13 years)
4	Karthik Madhavan, 2019 [19]	4	N/A
5	Traversi L, 2020 [41]	9	2005–2019 (14 years)
6	Ramírez-Huaranga, M.A., 2013 [42]	5	2008–2012 (4 years)
7	Aydın üNAL, 2017 [43]	9	2010–2016 (6 years)
8	Cassó-Troche L.R., 2022 [44]	11	2011–2012 (1 year)
9	Abid S., 2008 [17]	13	1997–2006 (9 years)
10	Chen, LH., 2010 [45]	16	1997–2006 (9 years)
11	Wong, S.S.; 2011 [46]	6	2000–2005 (5 years)
12	Kovalik, E C., 1996 [47]	10	1991–1996 (5 years)
13	Vinay Jain K., 2020 [48]	34	2014–2019 (5 years)
14	Mei-Yi Wu, [49]	12	2003–2006
15	Lu, Yueh-An, 2018. [50]	102	13 years
16	Yildirim S., 2022; [51]	15	N/A
17	Tsuchiya K., 2004 [52]	9	N/A
18	Faria B., 2011 [53]	11	5 years

**Table 10:** Antibiotic therapy according to identified bacterial sensitivity.

Article	Etiological Agent	Antibiotic	No. of Patients (%)	Treatment Length	Outcome
***Lu YA* (*2017*) ** [39]	*Enterococcus faecalis*	Ampicillin	1	35 d	Relapse
	*Enterobacter cloacae*	Vancomycin + Piperacillin/Tazobactam	1	42 d	Relapse
	*MSSA*	Cefazolin + Gentamicin	1	30 d	Recovered
	*Coagulase Negative Staphylococci*	Teicoplanin	1	25d	Death
	*MRSA*	Teicoplanin, Daptomycin, Teicoplanin + Rifampicin	1	115 d	Recovered
	*S. Epidermidis*	Vancomycin, Teicoplanin	1	53 d	Recovered
***Kuo G.* (*2018*) ** [16]	*MRSA*	N/A	30 (28.6)	N/A	N/A
	*MSSA*	N/A	9 (8.6)	N/A	N/A
	*Coagulase Negative Staphylococci*	N/A	14 (13.3)	N/A	N/A
	*Enterococcus*	N/A	7 (6.6)	N/A	N/A
	*Streptococcus spp.*	N/A	2 (1.9)	N/A	N/A
	*Klebsiella pneumoniae*	N/A	1 (0.95)	N/A	N/A
	*Candida parapsilosis*	N/A	1 (0.95)	N/A	N/A
	*Mycobacterium tuberculosis*	N/A	1 (0.95)	N/A	N/A
	*Mycobacterium chelonae*	N/A	1 (0.95)	N/A	N/A
***Traversi L* (*2020*) ** [41]	*Staphylococcus aureus*	Vancomycin + Gentamicin	1	4 w	Relapse
		Vancomycin + Ciprofloxacin + Ceftazidime	1	8 w	Recovered
		Vancomycin + Ciprofloxacin	1	8 w	Paraplegia
		Teicoplanin, then Linezolid	1	8 w	Recovered
	*Streptococcus agalactiae*	Vancomycin + Levofloxacin	1	4 w	Recovered
***Marco A.* (*2013*) ** [42]	*MSSA*	Vancomycin + Ceftazidime	1	N/A	Death
		Ceftazidime, Levofloxacin IV, then Levofloxacin and Rifampicin PO	1	1 m 6 w	Recovered
	*S. Epidermidis*	Vancomycin then Daptomycin	1	3 m + 3 m	Recovered
	*Enterococcus faecalis*	Vancomycin and Cefotaxime	1	N/A	Recovered
***Aydın Ünal* (*2017*) ** [43]	*S. Epidermidis*	Ampicillin + Sulbactam	1	N/A	Recovered
		Ampicillin + Sulbactam + Rifampicin	1	N/A	Recovered
		Teicoplanin	1	N/A	N/A
		Vancomycin + Meropenem	1	N/A	N/A
	*MSSA*	Vancomycin + Piperacillin/ Tazobactam	1	N/A	Death
***Kovalik*** **(*1996*)** [47]	*MRSA+MSSA*	Vancomycin	10	4–6w	
*Length of treatment* (*days*)*: mean/median/IQR: 54.53/42/26* *Length of treatment* (*days*) *for recovered patients 56/50/68.5*					

Abbreviations: MRSA—Methicillin-Resistant Staphylococcus aureus, MSSA—Methicillin-Sensitive Staphylococcus aureus, d—days, w—weeks, m—months, NA—not available, Staph E.— Staphylococcus epidermidis, IQR—interquartile range.

**Table 11:** Empiric antibiotic therapy combination when etiology was uncertain.

Nr.	Article	Empiric Treatment Scheme	Duration	No. of Patients	Outcome
1.	**Cervan AM (2012)** [38]	Mono or combined antibiotic therapy	iv (4 w), then oral (6w) after the clinical symptoms settled down	11	Death of 3 patients
2.	**Lu YA (2017)** [39]	Teicoplanin + Ceftriaxone	25 d	1	Death
		Teicoplanin + Ceftazidime, Teicoplanin + Imipenem/Cilastatin, Vancomycin + Flomoxef	38 d	1	Relapse
		Vancomycin	40 d	1	Recovered
		Vancomycin + Ceftriaxone	42 d	1	Recovered
		Vancomycin +Ceftriaxone	23 d	1	Recovered
		Vancomycin + Meropenem	55 d	1	Recovered
		Ceftriaxone, Vancomycin + Piperacillin/Tazobactam	18 d	1	Death
		Cefazolin, Oxacillin	24 d	1	Recovered
3.	**Traversi L.** **Nava E. (2020)** [41]	Vancomycin + Ciprofloxacin; then Teicoplanin + Ceftazidime	8 w	1	Recovered (2 m)
		Teicoplanin + Ciprofloxacin	4 w	1	Recovered (3 m)
		Levofloxacin + Rifampicin	4 w	1	Recovered (8 m)
		Ciprofloxacin	8 w	1	Recovered (3 m)
4.	**Marco A. Ramirez Huaranga (2013)** [42]	Vancomycin + Ceftazidime	4 w	1	Death
		Vancomycin + Ceftazidime	1 w	1	Treatment change (*Enterococcus* identified)
5.	**Aydın Ünal (2017)** [43]	Teicoplanin	N/A	1	Recovered
		Ampicillin + Sulbactam	N/A	1	Recovered
		Teicoplanin	N/A	N/A	Recovered
		Amoxicillin/clavulanic acid + Ciprofloxacin	N/A	1	Recovered
6.	**Chen, LH.** **(2010)** [45]	Cefazoline + Gentamicin	Over 6 w	5	N/A
		Vancomycin	N/A	2	N/A
*Length of treatment* (*days*)*: mean/median/IQR: 35,62/28/27.5* *Length of treatment* (*days*) *for recovered patients 39,16/39/30.75*					

Abbreviations: d—days, w—weeks, m—months, N/A—not available, IQR—interquartile range.

## Data Availability

The data presented in this study are available on request from the corresponding author.

## Supplementary Materials

The following supporting information can be downloaded at: https://www.mdpi.com/article/10.3390/antibiotics13030284/s1, Figure S1: Finally selected articles useful for our statistic.

## Abbreviations

ISD—infective spondylodiscitis; HD—hemodialysis, CKD—chronic kidney disease.
