# Supplementary material for: Antimicrobial Treatment Challenges in the Management of Infective Spondylodiscitis Associated with Hemodialysis: A Comprehensive Review of Literature and Case Series Analysis
Source: Antibiotics (Basel). 2024 Mar 20;13(3):284. doi: 10.3390/antibiotics13030284 (PMC10967641; doi:10.3390/antibiotics13030284)
Supplement: Supplementary file 1 [file antibiotics-13-00284-s001.zip › antibiotics-2877326-supplementary.pdf]

Supplementary Materials

Antimicrobial Treatment Challenges in the Management of Infective Spondylodiscitis Associated with Hemodialysis: A Comprehensive Review of Literature and Case Series Analysis

Ioana A. Ratiu [1,2], Corina F. Moisa [1,*], Laura Țiburcă [1,3], Edy Hagi-Islai [4], Anamaria Ratiu [4], Gabriel Cristian Bako [1,2], Cristian Adrian Ratiu [5] and Liana Stefan [1]

Faculty of Medicine and Pharmacy, University of Oradea, 1st December Square 10, 410073 Oradea, Romania; ioana.ratiu@didactic.uoradea.ro (I.A.R.); tiburca.lauraelena@student.uoradea.ro (L.Ț.); gabriel.bako@didactic.uoradea.ro (G.C.B.); lantal@uoradea.ro (L.S.)

[2] Emergency Clinical Hospital Bihor County, Nephrology Department, 12 Corneliu Coposu Street, 410469 Oradea, Romania

[3] Emergency Clinical Hospital Bihor County, Rheumatology Department, 12 Corneliu Coposu Street, 410469 Oradea, Romania

[4] Faculty of Dentistry, University of Medicine and Pharmacy "Iuliu Hatieganu" Cluj-Napoca, Victor Babeș Street 8, 400347 Cluj-Napoca, Romania; hagi.islai.edy@elearn.umfcluj.ro (E.H.-I.); ratiu.anamaria@elearn.umfcluj.ro (A.R.)

[5] Faculty of Medicine and Pharmacy, Dentistry Department, University of Oradea, 1st December Square 10, 410073 Oradea, Romania; ratiu_cristian@yahoo.com

* Correspondence: corinamoisa@hotmail.com

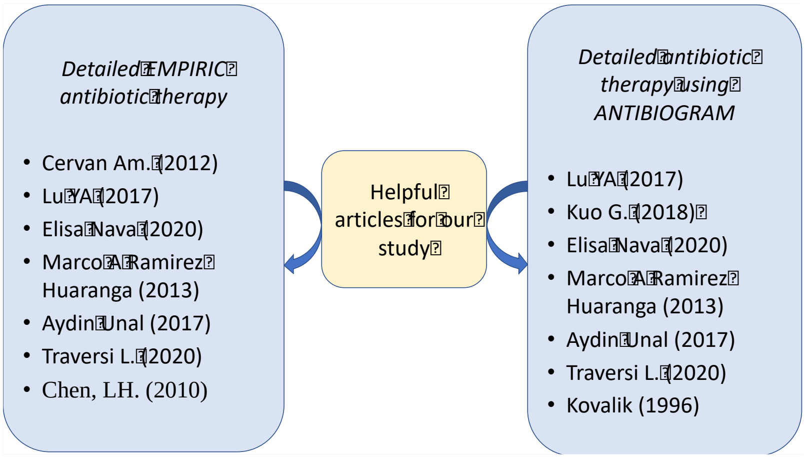

Figure S1. Finally selected articles useful for our statistic
